# Probing of three-dimensional spin textures in multilayers by field dependent X-ray resonant magnetic scattering

**DOI:** 10.1038/s41598-023-38029-5

**Published:** 2023-07-20

**Authors:** Erick Burgos-Parra, Yanis Sassi, William Legrand, Fernando Ajejas, Cyril Léveillé, Pierluigi Gargiani, Manuel Valvidares, Nicolas Reyren, Vincent Cros, Nicolas Jaouen, Samuel Flewett

**Affiliations:** 1 Synchrotron SOLEIL, L’Orme des Merisiers, 91192 Gif-sur-Yvette, France; 2 Unité Mixte de Physique, CNRS, Thales, Université Paris-Saclay, 91767 Palaiseau, France; 3 ALBA Synchrotron Light Source, Cerdanyola del Vallès, 08290 Barcelona, Spain; 4 Pontificia Universidad Católica de Valparaíso, Avenida Universidad 330, Valparaiso, Chile; 5 Present Address: University of Santiago de Chile, Avenida Víctor Jara 3493, Estación Central, Santiago, Chile

**Keywords:** Ferromagnetism, Magnetic properties and materials, Surfaces, interfaces and thin films, X-rays

## Abstract

In multilayers of magnetic thin films with perpendicular anisotropy, domain walls can take on hybrid configurations in the vertical direction which minimize the domain wall energy, with Néel walls in the top or bottom layers and Bloch walls in some central layers. These types of textures are theoretically predicted, but their observation has remained challenging until recently, with only a few techniques capable of realizing a three dimensional characterization of their magnetization distribution. Here we perform a field dependent X-ray resonant magnetic scattering measurements on magnetic multilayers exploiting circular dichroism contrast to investigate such structures. Using a combination of micromagnetic and X-ray resonant magnetic scattering simulations along with our experimental results, we characterize the three-dimensional magnetic texture of domain walls, notably the thickness resolved characterization of the size and position of the Bloch part in hybrid walls. We also take a step in advancing the resonant scattering methodology by using measurements performed off the multilayer Bragg angle in order to calibrate the effective absorption of the X-rays, and permitting a quantitative evaluation of the out of plane (*z*) structure of our samples. Beyond hybrid domain walls, this approach can be used to characterize other periodic chiral structures such as skyrmions, antiskyrmions or even magnetic bobbers or hopfions, in both static and dynamic experiments.

## Introduction

Multilayered magnetic materials composed of stacking of ultrathin films have been crucial in the most important nanomagnetism and spintronics developments over the past 30 years. In these systems, it is nowadays possible to engineer the properties of each layer by selecting their thickness, materials and the characteristics of the interfaces between these thin films. These modifications allow one to tune the overall magnetic properties such that the effective magnetic anisotropy, the saturation magnetization, and magnetic interaction between magnetic layers, gives rise to a very rich playground for the elaboration of specific magnetic properties. One of the most important developments in this area was the discovery of the interfacial Dzyaloshinskii-Moriya interaction (DMI)^1–7^. Thin films and multilayer systems with asymmetric magnetic/heavy metal interfaces with perpendicular magnetic anisotropy (PMA) give rise to interfacial DMI that favours cycloidal rotation of the magnetization around the DMI vector, thus favouring the emergence of chiral magnetic structures. The development of these DMI stacks has been essential in the discovery of magnetic systems hosting a rich variety of possible spatial orderings for their magnetization, being either isolated or periodic, called spin textures, such as chiral domain walls (DWs)^3, 8–10^, skyrmions^11–14^ and spin spirals^3, 8, 14^. In multilayers, in absence of chiral interaction, the dipolar energy might stabilize Néel DW configurations in the topmost and bottommost layers. As for the case of closure domains^15^, due to the direction of the stray fields, in the topmost layers the magnetization of the DWs hence rotate clockwise (CW), while in the bottommost they rotate counter-clockwise (CCW). On the contrary, a DMI strong enough to overcome the dipolar energy imposes a fixed chirality for the DW throughout the complete vertical direction. In the intermediate case, when DMI is not strong enough to overcome the dipolar energy in the topmost or bottommost layers, the multilayer DW will adopt an “hybrid chirality”^16–19^. Other energy terms, such as Zeeman energy from an external field, can then modulate the DW configuration.

In order to understand the properties of these spin textures, such as how robust they are against manipulation with external magnetic fields, and how they evolve and move due to electrical excitation, like spin transfer torque among others, it is necessary to have access to all three components of their spatially varying magnetization vector. While there are few techniques capable of doing so such as X-ray^20^, neutron^21^ or electron^22^ tomography, there are experimental restrictions that make these techniques limited in their applicability to decipher intricate and complex 3D magnetic structures, such as long acquisition time, and lack of X-ray facilities oriented to perform tomographic measurements, especially in a time resolved experiment, or under extreme experimental conditions. To overcome these restrictions, we perform field dependent X-ray resonant magnetic scattering (XRMS) measurements on multilayer systems containing chiral DWs, investigating the evolution of the DW profiles as a function of an in-plane external field strength. Moreover, we compare in detail these experimental results to micromagnetic and in-house developed XRMS simulations^23^, gaining access to a detailed description of the actual 3D spin textures in each layer using a fraction of beamtime needed for other techniques. We also resolve an important ambiguity remaining in our previous publication^23^, namely the inability to calibrate the true depth penetration of our X-rays due to the unknown amount of direct beam loss due to roughness and intercrystalline scattering before and after the reflection event under study. This is resolved interferometrically by incorporating theta-2theta curve measurements at incidence angles either side of the multilayer Bragg angle.

We have previously demonstrated the feasibility of determining the chirality of the topmost layers in DWs (CW or CCW)^24^ thanks to the magnetic asymmetry ratio at zero external field utilizing the circular dichroism of the XRMS signal. In this work, we extend this approach to study the evolution of the effective magnetic chirality under an applied external in-plane field in multilayers exhibiting hybrid chiral DW (HCDW)^18, 19^, which possess an internal magnetization which twists from Néel to Bloch, and often back to Néel along the direction normal to the sample’s surface. By studying the sign and intensities of the normalized circular dichroism (later named as asymmetry ratio) at different fields, we not only confirm the existence and influence of the Bloch-type part over the Néel-type part of the hybrid DWs but also show that we can determine the 3D magnetization spin texture by comparing the experimental results to micromagnetic and XRMS simulations.

### Sample fabrication and characterization

We configured our study using the following samples as listed in Table 1, chosen to exhibit different forms of hybrid chiral magnetization.

**Table 1:** Composition of the multilayer samples used in this work along the saturation magnetization $M_s$, the uniaxial anisotropy $H_k$ and the DMI constant value *D* used in the micromagnetic simulations.

Sample	Composition (thickness in nanometers)	$M_s$ (kA m$^{-1}$)	$\mu _0H_k$ (mT)	*D* (mJ m$^{-2}$)
S1	$\vert \vert$Ta(10)$\vert$Pt(8)$\vert$[Co(0.8)$\vert$Al$_2$O$_3$(1)$\vert$Pt(1)]$_{\times 20}\vert$Pt(2)	1373	358	0.9
S2	$\vert \vert$Ta(10)$\vert$[Al$_2$O$_3$(1.0)$\vert$Co(1)$\vert$Pt(1.0)]$_{\times 20}\vert$Pt(7)	1278	84	$-1.7$
S3	$\vert \vert$Pt(10)$\vert$[Ir(1.0)$\vert$Co(0.8)$\vert$Pt(1.0)]$_{\times 5}\vert$Pt(3)	1229	640	$-2.0$

**Figure 1:**
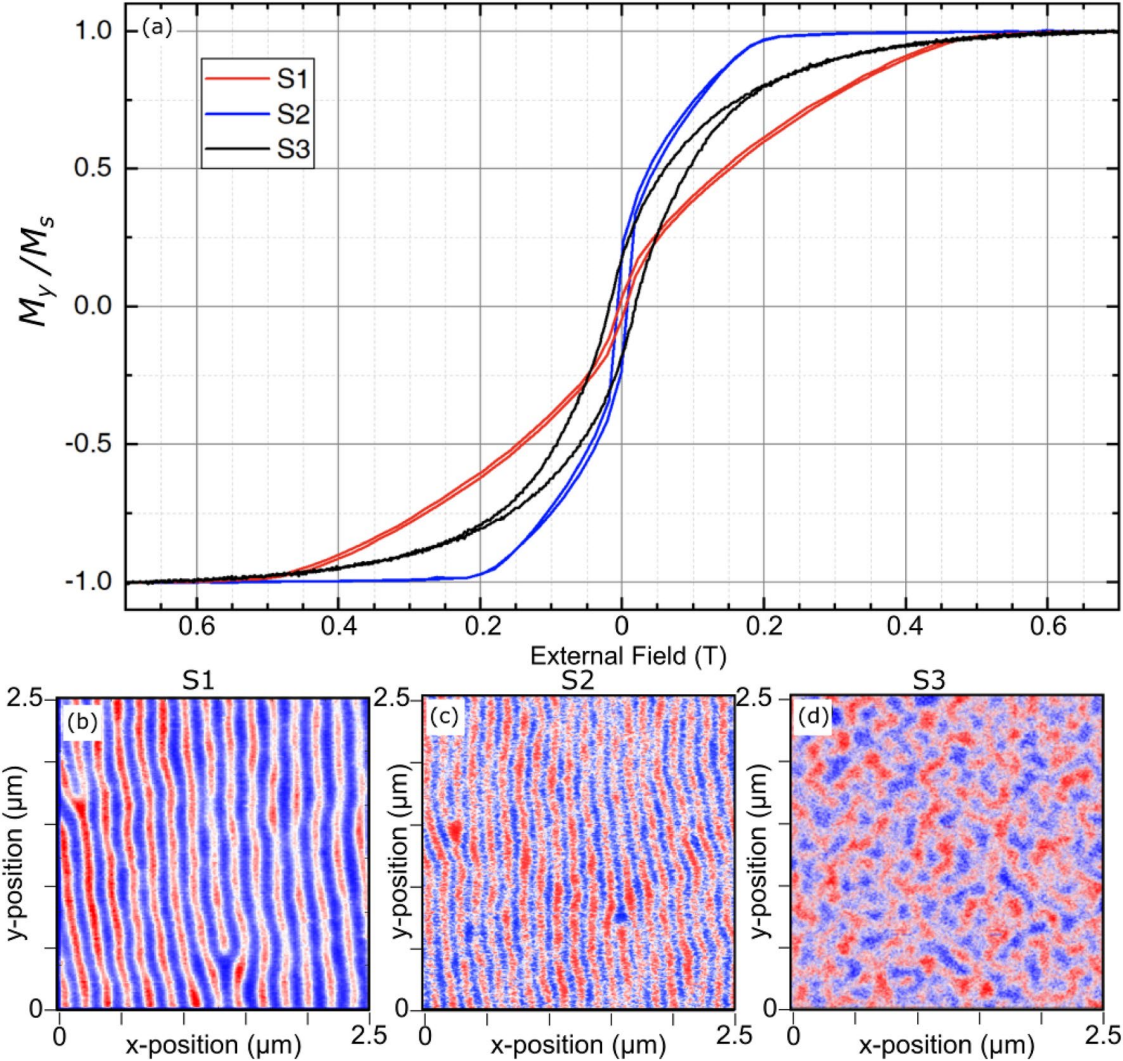
Magnetization hysteresis and zero-field MFM measurements for the three samples used in this work (sample details presented in the main text). (**a**) Hysteresis curves, in the hard plane (sample plane), normalized by the saturation magnetization Ms of each sample. (**b**–**d**) MFM phase maps for samples S1, S2 and S3 respectively. The colour codes the attraction of the magnetic tip (phase signal), and reveals the domain structure. The period is easily determined from such MFM images.

**Figure 2:**
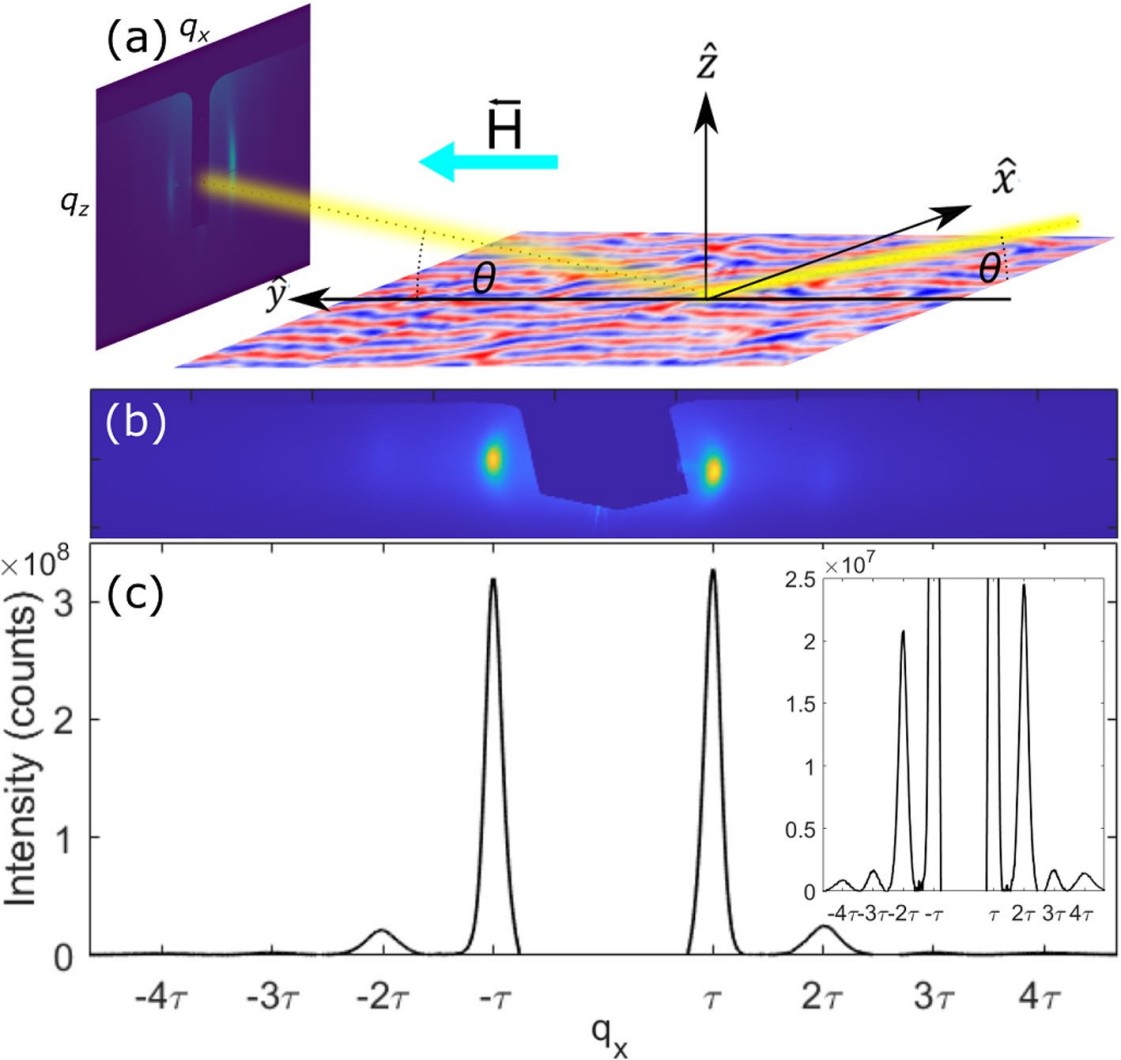
(**a**) Description of the XRMS experimental measurements. X-rays (yellow) incident on the sample at a $\theta$ angle. The diffraction pattern arising from the interaction between the X-rays and the magnetic configuration are captured in the CCD camera. External magnetic field is applied parallel to the X-rays incidence plane, along the $\hat{y}$ direction. The sample surface, an actual MFM phase map, displays domain configuration: “up” and “down” domains are colour-coded in red and blue. (**b**) Example of a unpolarized XRMS pattern (obtained by summing circular left and circular right polarization images), for sample S1 under an in-plane magnetic field of 0.175 T. (**c**) Profile of the XRMS shown in (**b**) along $\hat{q_x}$ direction after removing the background. We calculated the averaged sum of the column values of CCD pixels in (**b**) to obtain a value assigned to the $q_x$ position of that column. Inset in (**c**) shows a zoom of the *y* axis of (**c**) to better evidence the 3$\textrm{rd}$ and “4$\textrm{th}$” order.

All experiments and measurements were performed at room temperature. Our strategy in terms of sample choice was to compare two samples with similar dimensions and magnetic domain structures, S1 and S2, each comprising 20 repetitions of magnetic and non-magnetic layers with opposite stacking sequence, and potentially hosting HCDWs. These two samples were then compared with a control sample S3 hosting a single chirality throughout the multilayer. The choice of an opposite order of the stacking between them allows the sign of the DMI constant to be reversed. We selected the sample S2 stack because it displays a stripe periodicity similar to S1, facilitating the comparison of the XRMS diffraction patterns between the reversed stacks S1 and S2. With the domain wall energy and the domain period being controlled by the anisotropy and the DMI, we had to use a reduced PMA in S2. The PMA is reduced both by the weaker (111) texture and the larger thickness of Co (see Table 1). Sample S3 is composed of [Ir(1.0)$\mid$Co(0.8)$\mid$Pt(1.0)]$_{\times 5}$ with a negative total DMI constant, large enough to impose the stabilization of a CW Néel DW uniform along the $\hat{z}$ direction of the multilayer stack (the coordinate system is drawn in Fig. 2a). Samples were characterized by alternating gradient field magnetometry (AGFM) and magnetic force microscopy (MFM), with hysteresis and zero-field MFM results shown in Fig. 1.

Sample S1 and S2 exhibit stripes periods, which are obtained by sample demagnetization using in-plane field as shown in Fig. 1a, the magnetization loops of S1 and S2 display different saturation fields in the hard plane, associated to different effective out-of-plane anisotropy. The associated stripe period is accordingly shorter for the sample with the smallest anisotropy and the largest DMI amplitude. Details of the fabrication and characterization of the samples can be found in the “Methods” section.

### XRMS measurements

In Fig. 2a we show a scheme of our experimental setup. XRMS measurements for sample S1 and S2 were performed in reflectivity mode both on SEXTANTS beamline^25^ at the RESOXS end-station^26^ on SOLEIL Synchrotron and for all samples on MARES end-station of the BOREAS beamline^27^ at ALBA synchrotron and, using X-rays of left (CL) and right (CR) circular polarization tuned to the L$_3$ Cobalt edge. An external in-plane field with a maximum value of 1 T is applied in order to study the values of XRMS diffraction peaks within a closed loop of the hysteresis curve.

Figure 2b is an example of an XRMS image for sample S1 under an in-plane magnetic field ($\mu _0H_{\text {ip}}=0.175$ T) along the $\hat{y}$ axis. The image has been geometrically corrected to account for the projection due the angular incidence of the X-rays. The sample-detector distance at ALBA is approximately 0.40 m, compared with 0.26 m at SOLEIL. All samples were measured at the L$_3$ Co edge energy of x-rays, which nominally corresponds to 777.6 eV. With the SOLEIL geometry and the CCD detector size, $q_x$ values up to $210\,\upmu$m$^{-1}$ are probed (taking $q_x=0$ at the centre of the CCD detector), allowing us to observe up to “4$\textrm{th}$” order diffraction peaks, as shown in Fig. 2c. ALBA geometry allowed us to measure $q_x$ values up to $137\,\upmu$m$^{-1}$. In order to perform an accurate comparison between the diffraction peaks caused by the stripe-domain configuration for different applied fields, we took all the values of one column of matrix signal given by the CCD, we summed them all in the $q_z$ direction, and we divided that sum by the number of values (in this case pixels) in that column. That value, the averaged sum of the column values of matrix signal given by CCD, was then assigned to the pixel position of that column in the $q_x$ direction. We repeated this calculation for each column producing a curve containing information about the intensity of the peaks and the background diffuse scattering. We removed the background diffuse scattering for each diffraction pattern obtained with circular left polarization ($I_\mathrm{CL \,}$) and circular right polarization ($I_\mathrm{CR \,}$), producing a clean curve as the one shown in Fig. 2c, for each helicity. We then summed, subtracted, and calculated the asymmetry ratio using these profiles, and we took the maximum intensity value of the relevant peaks.

## Simulations

### General description

In order to corroborate our assessment on the nature of the XRMS peaks and their behaviour under external field, we performed XRMS simulations using the protocol of Flewett et al.^23^ and micromagnetic calculations as input, with full details to be found in the “Methods” section. The simulation protocol^23^ was developed in parallel with this manuscript using the data presented here as a “training dataset” for fine tuning of the protocol.

Starting from a binary stripe domain pattern over a 2D space of either perfect or disordered stripe-domains, we inserted the domain wall profiles calculated by micromagnetic simulations for each field strength, evaluating the reflection coefficients over each of the 40 magnetic/non-magnetic interfaces present in the samples S1 and S2. If we were to simply propagate the phase matched reflected beams of amplitudes determined by the depth-attenuated reflection coefficients, we found that the observed behaviour of the first order peaks is reproduced in a satisfactory manner, but not so for the “second order peaks”. In order to reproduce the observed dichroism in the “second order peaks” it was necessary to propagate the incident and reflected beams through the magnetically heterogeneous sample, modulating the incident and reflected beams propagating parallel to the Bloch walls by the differential contrast produced by these domain walls. As such, what we present in this paper is not exclusively a reflection geometry experiment, but can be considered instead a hybrid of reflection and transmission geometries.

The imaginary part of the X-ray scattering factors was calculated from X-ray absorption spectroscopy measurements on a pure cobalt sample during beamline calibration, and calibrated by comparing with tabulated values of Henke et al.^28^ in the off-resonance limit. From here, the real parts were calculated by means of the Kramers-Kronig transformation. The exact values of the atomic scattering factors used in this paper were taken by matching the observed zero-field asymmetry ratio for S1 with the results from an energy scan on a sample identical to S1 as published in Fig. 8 of Ref. ^23^.

**Figure 3:**
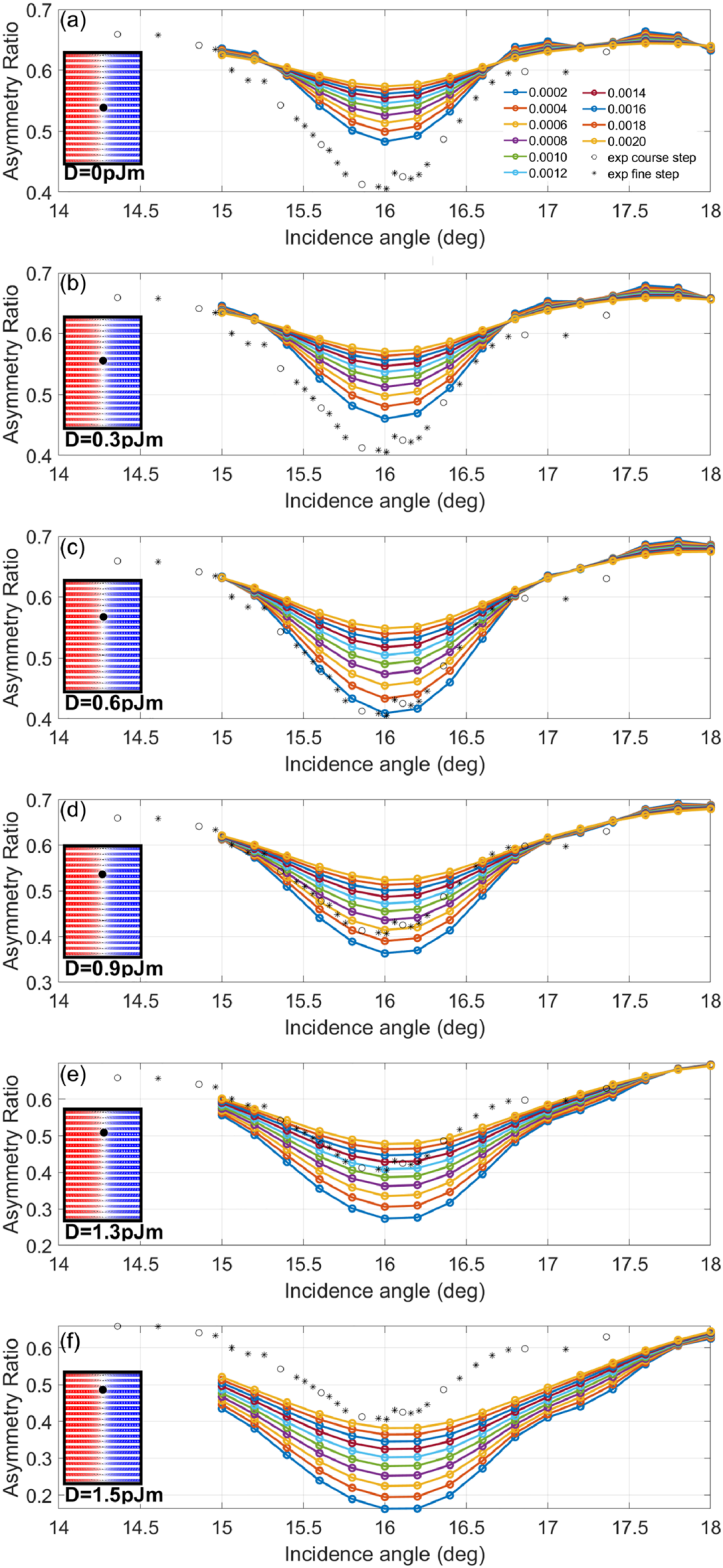
Experimental and simulated first order asymmetry ratios for a theta-2theta curve series made on S1 two degrees either side of the multilayer Bragg angle for this sample. In the inset in each subplot corresponding to a different value of the DMI, the expected chirality crossover is shown with a black dot, and each solid simulation line in the main figure of each subplot corresponds to a different value of $\Delta \beta$ as used in the simulation. Finally, the dotted line in each subplot corresponds to the experimental results. Readers should note how the FWHM of the dip increases with increasing DMI, corresponding to a chirality crossover closer to the surface.

### Theta-2theta curve curve calibration

In our previous publication concerning the development of the simulation algorithm utilized here^23^, an key weakness was our inability to determine the effective direct beam X-ray penetration depth due to the contribution of sample roughness and intercrystalline scattering. To compensate, in our previous work we introduced a “fudge factor” $\Delta \beta$, with which the imaginary part of the refractive index was increased, estimating this value based upon the expected crossover from positive to negative chirality according to the estimated value of the sample DMI as measured elsewhere^18^. Traditionally, XRMS measurements have been performed at the multilayer Bragg angles in order to maximise the signal to noise ratio, however this has come at the cost of the phase diversity present at incidence angles where the contributions from each layer do not necessarily sum in phase. In our previous publication^23^, we included one measurement of S1 performed at an incidence angle one degree removed from the Bragg angle, finding surprisingly that the first order magnetic asymmetry ratio jumped around 20 percentage points (alongside the disappearance of the second order dichroism). By performing a fine theta-2theta curve scan near to the Bragg angles, we find that the sharpness and depth of the reduction in 1st order asymmetry ratios on the multilayer Bragg peaks is strongly related to the real depth profile of the domain wall chirality within the sample. In Fig. 3 we show our experimental theta-2theta curve data for S1 compared with simulations where the increment in the absorption $\Delta \beta$ is varied for a domain wall profile drawn from micromagnetic simulations performed using different values of the DMI. In all these simulations the values of the uniaxial anisotropy and exchange stiffness as stated in Table 1 were used. From these results, it can be seen that the best fit for $\Delta \beta$ is found around 0.0008, with D = 0.9 mJ m$^{-2}$, close to the value determined by BLS of 1.0 mJ m$^{-2}$ for the same sample in a previous publication^18^. For S2 and S3, we assumed that $\Delta \beta$ takes the same value as for S1, given similar sample preparation conditions. Judging from Fig. 3, one can estimate the uncertainty on the DMI to be ± 0.3, and the uncertainty on $\Delta \beta$ to be ± 0.2. On the other hand, the value of the DMI has been constrained by Brillouin light scattering for these samples to a precision of +/− 0.1.

This calibration begs the question, “Is it possible to use XRMS as a tool for measuring the DMI by finding the depth of the crossover point in the domain wall chirality?” Answering this will require investigation of the relationship between the DMI and the chirality profile as first studied by Legrand et al.^18^, alongside studies of the the sensitivity of XRMS for determining the crossover point for a range of different sample types, alongside comparisons with BLS. Our results for S1 suggest that the answer to this question could well be “yes - with limits”, with the practical limits to be determined. For negative DMI values as is the case for S2, we expect such measurements to be far less sensitive due to the chirality crossover being buried deep within the sample. Our group is also actively investigating the degree to which this incidence angle dependent data can be used to resolve more complex 3D structure within magnetic multilayers, beyond the simple stripe domains studied in this paper.

## Results and analysis

### Basic results

**Figure 4:**
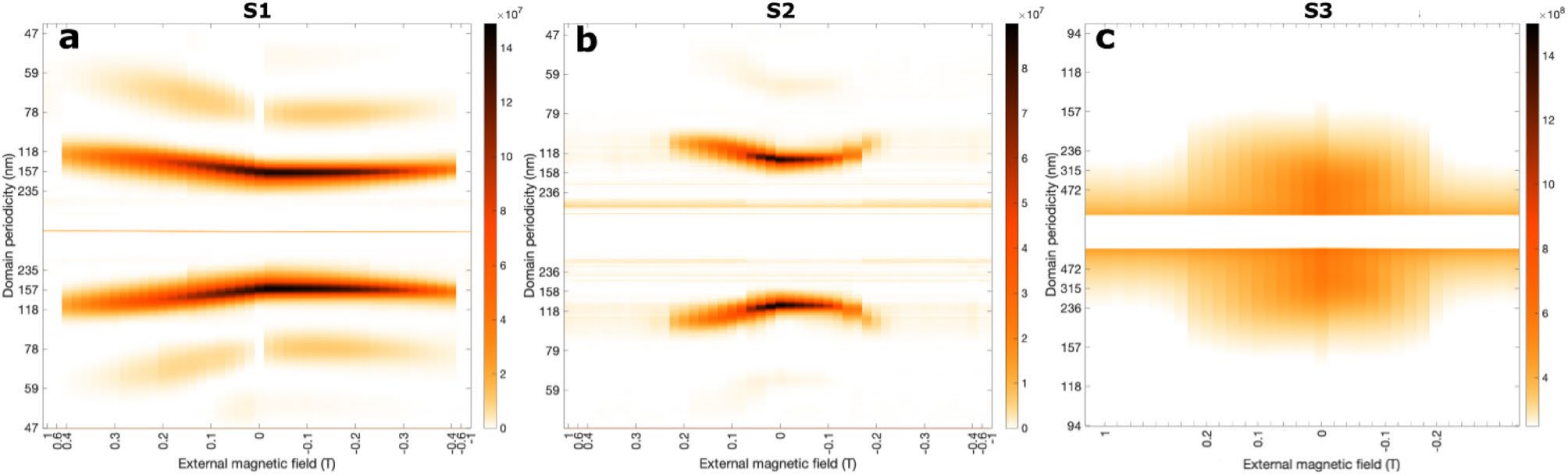
Scattered intensity (sum of both polarization) under an external field in plane ranging from 1 to − 1 T for sample (**a**) S1 and (**b**) S2, and (**c**) from 1 to − 0.8 T for sample S3. We have used a logarithmic colour scale to better display the “second order peaks”. Note that for (**c**) the horizontal axis markers are the following: 0.1 T steps from 1 to 0.2 T, 0.025 T steps from 0.2 to 0.1 T, and 0.02 T steps from 0.1 to 0 T, same for the negative fields.

In Fig. 4 we show the background subtracted scattered intensity ($I_\mathrm{CL \,} + I_\mathrm{CR \,}$) for one direction of the external magnetic field sweep applied to all samples. The first order peak (FOP) is ascribed to the diffraction from the out-of-plane domain configuration, for which the periodicity can be derived from their position in the $q_x$-direction: scattering peaks are located symmetrically around the specular spot (blocked by a beamstop) at $\delta q_x = \pm \, \tau$, where $t = 2\pi /\tau$ is the domain periodicity^15^. At zero field, we determine a magnetic period of 165 nm, 125 nm and 250 nm for sample S1, S2 and S3 respectively. The dispersion in period associated with these values is $\sim 10\%$ estimated from the full width half maximum of the FOPs. Readers should note that the FWHM values are greater at positive than at negative fields, suggesting a higher degree of stripe disorder as the field is being reduced from saturation at + 1 T towards zero, and a greater degree of stripe order at negative fields applied during field reversal.

**Figure 5:**
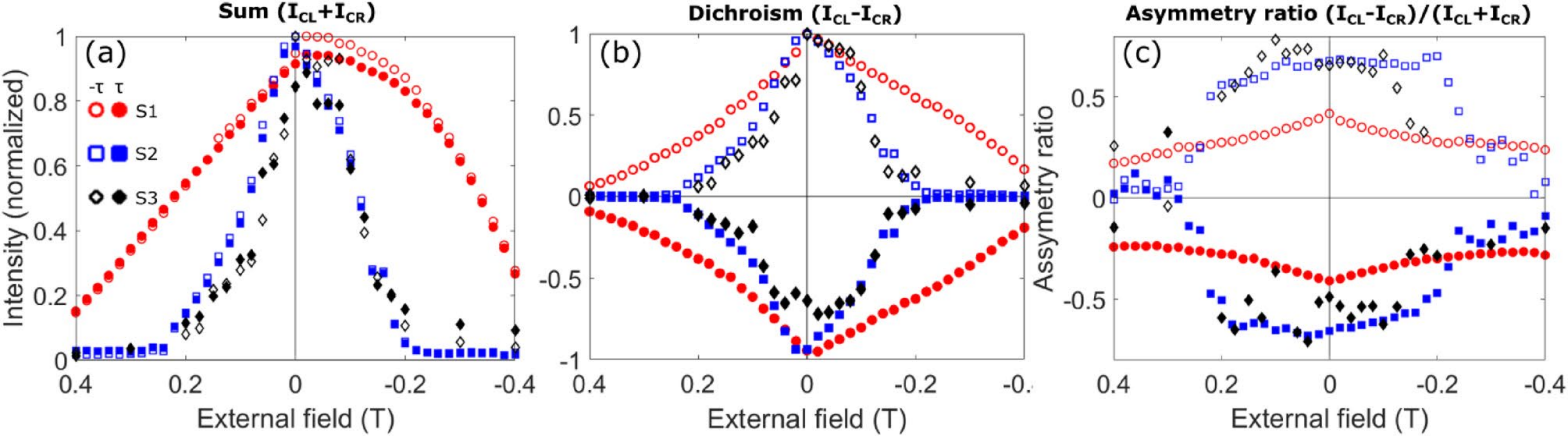
First order peak intensity under an in-plane magnetic field. (**a**) The normalized sum, (**b**) normalized dichroism, and (**c**) asymmetry ratio of the left and right circular polarization XRMS first order peaks (FOPs) for sample S1 (red, circles), S2 (blue squares) and S3 (black, diamonds) respectively. Open (filled) symbols represent the FOP intensity at $- \,\tau$ ($+\,\tau$). In (**a**)–(**c**) the field scale is inverted to emphasize the temporal sequence from left to right.

### Discussion of XRMS under an in-plane magnetic field

In Fig. 5 we display the main experimental results of our XRMS study. We display the FOPs of the summed intensity ($I_\mathrm{CL \,}$+$I_\mathrm{CR \,}$) in Fig. 5a, the dichroism ($I_\mathrm{CL \,}-I_\mathrm{CR \,}$) in Fig. 5b and the asymmetry ratio ($I_\mathrm{CL \,}-I_\mathrm{CR \,}$)/($I_\mathrm{CL \,}$+$I_\mathrm{CR \,}$) in Fig. 5c, for fields ranging from 0.4 to $-\,0.4\,\text {T}$ and for samples S1 (red, circles), S2 (blue, squares) and S3 (black, diamonds). Open symbols represent the peaks located at $-\, \tau$ in *q*-space, while filled symbols indicate peaks at $+ \,\tau$. All samples’ XRMS are studied at the first Bragg peak (close to 16°) of the multilayer, where the circular dichroism is maximum. Despite this low incidence angle, X-rays are still probing the texture down to the Bloch part of the thick multilayers S1 and S2—with the coherent addition of the reflections from each layer playing a role in augmenting the effective penetration of the X-rays^23^. In order to compare the behaviour of the peaks’ intensities for the sum and the differences of $I_\mathrm{CL \,}$ and $I_\mathrm{CR \,}$ between samples, we normalize each set of data by the maximum peak value between the FOPs at $\pm \tau$ for each sample. Before these measurements, the magnetization was saturated by applying 1 T in-plane direction along $\hat{y}$ axis, i.e., parallel to the X-ray propagation projected on the sample plane.

A clear outcome from the analysis of the peak intensities in Fig. 5a is that the peak intensity profiles of samples S2 and S3 differ from that in sample S1 when the field is swept from 0.4 to $-\,0.4\,$T. While samples S2 and S3 have a similar decay of the intensities around the maximum, sample S1 presents a markedly different distribution of the intensities around its maximum near zero field. A similar behaviour can be also observed in Fig. 5b. These differences, which are related to the sensitivity of the x-rays to the magnetic configuration of these domain walls close to the surface, will be discussed later on.

In Fig. 5c, we present the asymmetry ratio in which we observe that both peaks’ ($\pm \tau$) signs remain opposite for all fields for which a non-zero XRMS signal is measured. As shown before^15, 18, 24, 29^, the position and signs of these peaks in the ($q_x$,$q_y$) plane determine the periodicity and sense of rotation (chirality) of the DW chiral texture within the penetration length sensed by X-rays, which for all the studied samples indicates CW Néel DW. With these results we may conclude that the chirality of the Néel domain walls is not altered as a result of sweeping the field.

**Figure 6:**
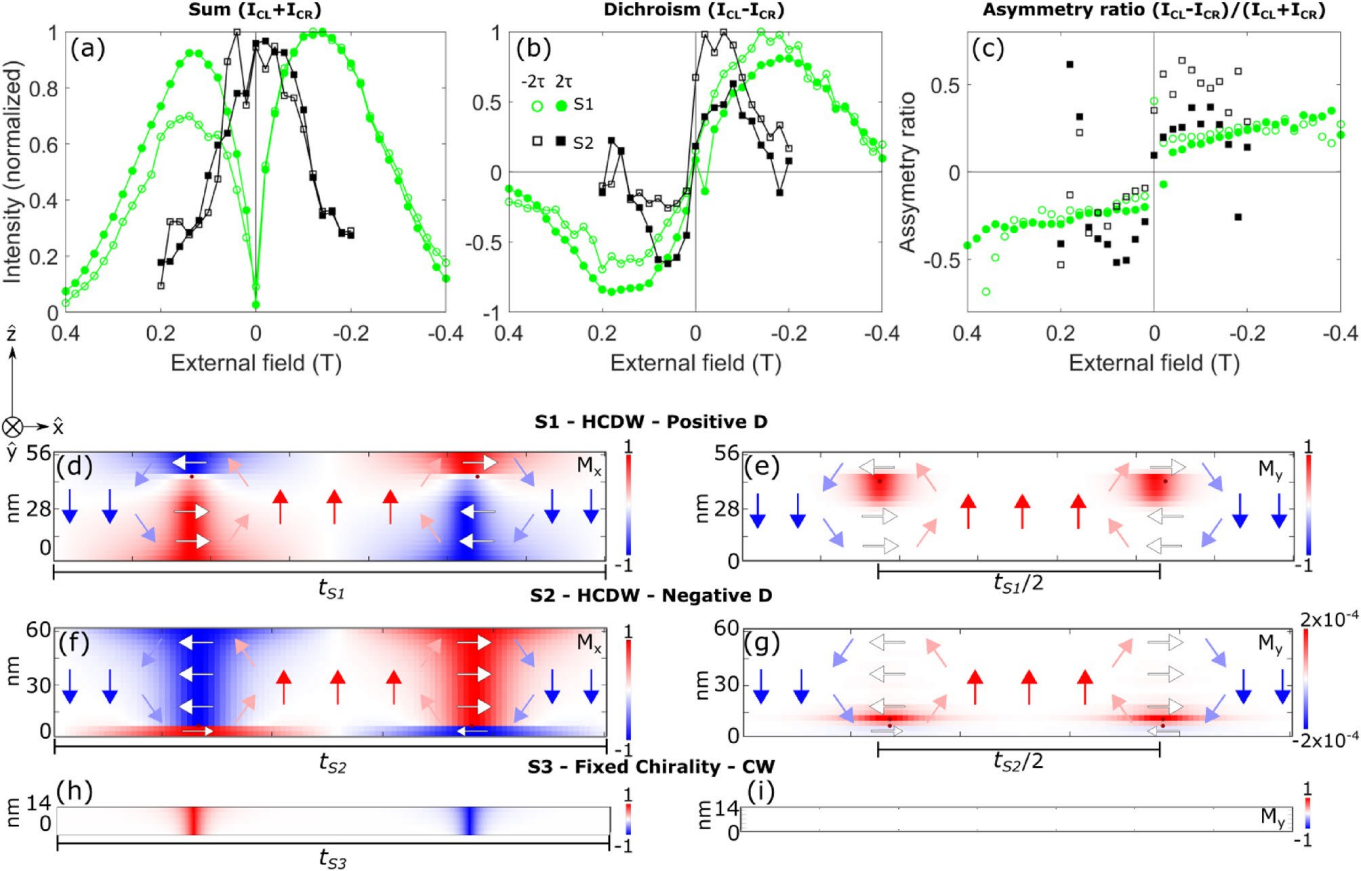
“Second order peak”intensity under an in-plane magnetic field. (**a**) The normalized sum, (**b**) normalized dichroism, and (**c**) asymmetry ratio of the left and right circular polarization XRMS “second order peaks” (“SOPs”) for sample S1 (green, circles), S2 (black, squares) respectively. Open (filled) represent the “SOP” at $-\, 2\tau$ ($+\,2\tau$). Data points are joined as a eyeguide. (**d**)–(**i**) Transversal cut along $\hat{x}$ of micromagnetic simulations showing the $\hat{x}$ and $\hat{y}$ (background colour) and $\hat{z}$ (arrows) components of the magnetization for S1 [(**d**, **e**)], S2 [(**f**, **g**)] and S3 [(**h**, **i**)] respectively. We plot only the magnetization angle in the DW of each magnetic layer without considering the spacer and heavy metal layers. The colourbar in (**g**) has been modified to increase the contrast of $M_y$ values for a better visualization. The magnetic states are shown at zero field after being saturated at 1 T in $\hat{y}$ direction. Domain walls are spontaneously generated at random *x* position and are aligned for convenience. Points at 0 and 0.19 T are also shown in Flewett et al.^23^.

In these XRMS experiments, we interestingly also detect what we ascribed to be “second order peaks” (“SOPs”) in samples S1 and S2 but not in S3, and our modelling suggests that their presence is due to field-aligned Bloch walls generating a periodic structure with half the period of the chiral structure responsible for the first order peaks. Therefore this peak is the first order peak generated by the periodic structure of the field-aligned Bloch walls. Even though the rigorous definition of a second order in a diffraction pattern does not completely fit the nature of this peak, we have called them “SOPs” for the sake of a better understanding of our work. In Fig. 6a–c, we display the “SOPs” for the summed intensity ($I_\mathrm{CL \,}$ + $I_\mathrm{CR \,}$), dichroism ($I_\mathrm{CL \,}$ – $I_\mathrm{CR \,}$) and the asymmetry ratio ($I_\mathrm{CL \,}$ – $I_\mathrm{CR \,}$)/($I_\mathrm{CL \,}$ + $I_\mathrm{CR \,}$) for samples S1 and S2. The position of these peaks in *q*-space indicates a periodic structure with a periodicity half that of the out-of-plane magnetic domains ($\frac{t}{2}$). This is in accordance with the periodicity of the in-plane magnetization ($M_{\text {y}}$) within the HCDW. In Fig. 6d–i we present an example of the magnetization profiles calculated by our set of micromagnetic simulations for samples S1, S2 and S3 at zero field. These transversal cuts show the magnetization components (background colour, red and blue) along ${\hat{x}}$ ($M_\text {x}$, left set) and ${\hat{y}}$ ($M_\text {y}$, right set), which are in agreement with previous results in these multilayers at zero field^18, 19^.

The “SOPs” present notable differences compared with the signal of the FOP. The summed signal (Fig. 6a) decreases its intensity near zero field, almost vanishing for sample S1. A similar behaviour is expected on sample S2, and we do observe a small decrement of the signal near to zero field, but the resolution of the steps between external fields did not allow us to observe this decreasing in intensity as clearly as for sample S1. This shows that we are observing hysteresis by XRMS and meaning that “SOPs” just appear when the in-plane field is applied. Additionally, these peaks are dichroic in both samples, S1 and S2 (Fig. 6b), that is, one circular polarization of the X-rays produces a stronger signal than the opposite. However, we notice that the “second order” dichroic contrast has the same sign between peaks at $\pm \,2\tau$ and switches sign as the external applied field passes through zero, in contrast to FOPs for which the dichroism changes sign between peaks at $\pm \,\tau$, which is a consequence of the chiral nature of the magnetic domains. Hence, we conclude that the “SOP” dichroic contrast is not directly related to the effective chirality of the probed textures, but rather the periodic structure of aligned Bloch walls. In these results, the $2\tau$/$-\,2\tau$ differences observed in reciprocal space - especially in the case of S1 are believed to be due to disorder present in the stripe structure. At positive field during which the stripes are forming, this $2\tau$ and $-\,2\tau$ asymmetry is present, however it is not observable at negative field where the stripes exhibit a greater degree of spatial order. The field dependence of the sum, dichroism and asymmetry ratio values are related to the changes of the in-plane magnetization (Bloch part of the DW) with the field, in both samples, being these effects more noticeable in sample S1 than S2 due to the position of the Bloch part of the DW closer to the surface of the sample, as it will be explained in the next section.

**Figure 7:**
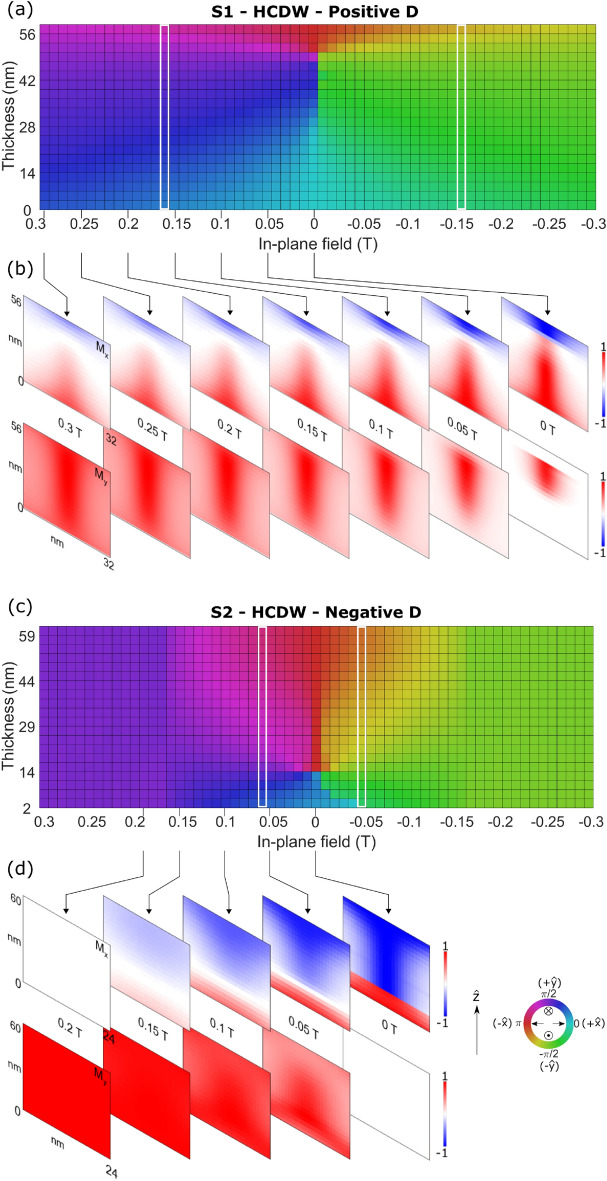
Azimuthal angle of the magnetization at each layer (rows) along the vertical direction (columns) of a single domain wall. Each column represents each field step between 0.3 and − 0.3 T for sample (**a**) S1 and (**c**) S2. The angles were calculated using micromagnetic simulations. The direction of the in-plane magnetization associated to the colour scale is graphically displayed by the inner symbols of the colour wheel. White rectangles point the external in-plane field at which the top layer angle is at 45° from pure Néel or pure Bloch texture. We plot only the magnetic layer’s magnetization without considering the spacer and heavy metal layers. The (**b**) and (**d**) panels show snapshots of the $M_{\text {x}}$ and $M_{\text {y}}$ components of the magnetization at different fields for S1 and S2 respectively.

### Consequences of Bloch-type DW position within the HCDW on XRMS peaks

We explain the difference in the behaviour of the FOP intensities between S1, S2 and S3 as a signature of the change of the vertical profile (along $\hat{z}$) of the magnetization within a domain wall when an in-plane field is applied. Micromagnetic simulations performed for samples S1, S2 and S3 confirm that as soon as the stripe-domain phase is formed, DWs for samples S1 and S2 present hybrid chirality which, on the contrary, is not observed in sample S3 (see Fig. 6e–h).

The initial magnetization of our samples is saturated in ${\hat{y}}$ direction and when the external field is decreased to $\sim$ 0.45 T (for S1) or $\sim$ 0.22 T (for S2) a stripe-domain phase first appears, giving rise to the “SOPs”. Our analysis reveals that under this value, the orientation of the magnetization in the inner region of each DW is determined by the direction of the applied field along ${\hat{y}}$, thus forming Bloch-type DW, together with small Néel-type DWs portions located at the top and bottom layers. When the in-plane field is reduced towards zero, the Néel-type DW of either chirality becomes increasingly predominant and, at zero field, the DW is mostly a Néel DW with a small Bloch-type DW section aligned with the previous direction of the applied field. The position of this Bloch-type DW section along the $\hat{z}$ direction of the multilayers depends on the sign and value of *D*^18^. When reversing the in-plane field, the Bloch DW type reverts its magnetization, always following the external magnetic field direction, and starts to grow in size along the ${\hat{z}}$ direction, finally reaching the topmost and bottommost layers.

This behaviour of progressive DW reorientation can be observed in Fig. 7a and c in which we plot the azimuthal angle of the magnetization within one domain wall for all layers of samples S1 and S2 respectively, for all field steps between 0.3 and $-\,0.3$ T. This illustrates which magnetic component of the DW, Bloch or Néel, is more relevant in each layer along ${\hat{z}}$ direction. In Fig. 7b and d we show some snapshots of the $M_{\text {x}}$ and $M_{\text {y}}$ components of the DW magnetization at different fields to better understand the changes of the DW magnetization with the applied field. Since the Bloch DW grows in the ${\hat{z}}$ direction when the in-plane field is increased, reaching the topmost layers where X-rays are more sensitive to the magnetization, we ascribe the peak intensities asymmetry seen in Fig. 5a and c around the peak’s maximum to the field induced Néel-Bloch transition. As the field is applied, the modulation in $M_z$ is reduced from one domain to the other, reducing the intensity of the 1$\textrm{st}$ order scattering signal. What grows in contrast is the $M_y$ contribution to the scattering signal which manifests itself in the specular, “2$\textrm{nd}$” and “4$\textrm{th}$” order peaks.

In the case of sample S1, the in-field behaviour of the “SOPs” produced by the in-plane periodic magnetization modulation can be described as follows: when in-plane external field is swept from 1 T down to  0.5 T the sample magnetization remains saturated and therefore no XRMS signal is found. When the field is further decreased from 0.5 T to $\sim 0.16\,\text {T}$ for sample S1 ($\sim \,0.06\,\text {T}$ for sample S2), disordered quasi-periodic stripe domains form, producing broadened XRMS peaks. The Bloch-type DW is predominant over the Néel-type DW magnetization inside the magnetic top layers (see Fig. 7a and c), explaining that the “SOP” dichroic peak reaches maximum at this value (see Fig. 6b). After this maximum, Néel-type DW magnetization is favoured against the Bloch-type DW magnetization at the topmost layers decreasing these peaks’ intensities until being only weakly present at zero field and vanishing at around − 20 mT for S1—just after field reversal. The same explanation can be applied for sample S2, whose Bloch DW has the same behaviour with the in-plane field, but is buried deeper in the multilayer stack ($M_{\text {y}}$ in Fig. 6e and g). As a consequence, the maximum “SOP” summed intensity for S2 is 6 times smaller compared to S1 “SOP” maximum summed intensity.

**Figure 8:**
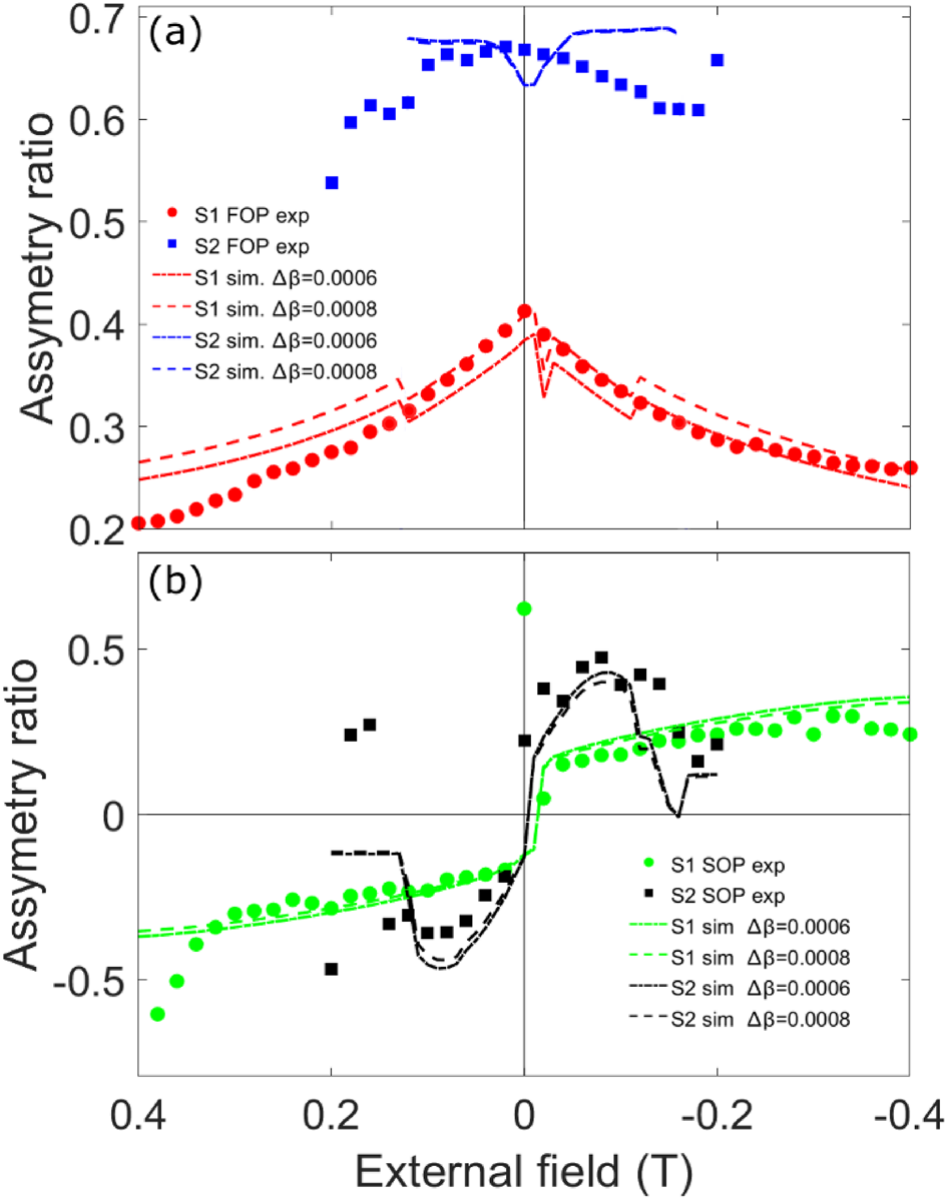
Averaged values of the asymmetry ratio peaks for (**a**) $\pm \tau$ (FOP) and (**b**) of $\pm 2 tau$ (“SOP”) for sample S1 (red and green) and S2 (blue and black). Dashed lines were calculated using XRMS simulations based on micromagnetic calculations for each field step and using two different $\Delta \beta$ values.

### Comparison of experiment and simulation

To compare the experimental and simulated results, we have averaged the values of the asymmetry ratio (previously shown in Figs. 5c and 6c) for both peaks $\pm \tau$ as is shown in Fig. 8. We note that within the range below the saturation magnetization, the experimental and theoretical asymmetry ratios agree to within a window of +/− 0.1, a figure which increases as one moves towards saturation where the magnitude of the magnetic modulations giving rise to the asymmetric scattering tends towards zero. The good agreement between experiment and simulation for both peak orders for sample S1 is an indication of the accuracy of the micromagnetic simulations representation of the real in-depth magnetization of sample S1, along with a robust XRMS simulation. Readers will observe the presence of a jump in the simulated asymmetry ratios at around 0.1 T for S1. This is due to the discretization of the sample in the *z* direction, and the chirality crossover moving from the 5$\textrm{th}$ to the “4$\textrm{th}$” layer from the top at this field value (out of the 20 layers in the sample).

In a similar way, sample S2 XRMS simulations are also in close agreement to the experimental data when the in-plane field is close to zero. However due to its small coercive field compared with S1 and the fast change of the magnetic domain periodicity under field, experimental and simulated data is not in such good agreement when the external in-plane field grows larger than $0.05\,$T.To further improve the quality of the match between simulation and experiment, it would be ideal to have on hand a field-dependent real space map of the domain pattern in order for the stripe disorder to be incorporated correctly in the simulation. The generally good agreement between experiment and simulation observed here confirms the applicability of the domain wall profile as proposed by the micromagnetic simulations, with the micromagnetic simulations in turn being backed up by consistency between the parameters used and the experimental scattering data.

## Summary

In this work we performed XRMS measurement on multilayer samples engineered to contain Hybrid Chiral Domain Wall (HCDW) under an in-plane magnetic field regime. We have determined the magnetic asymmetry ratio and we studied its intensity evolution as a function of the external magnetic field, using micromagnetic and XRMS simulations to draw conclusions about the resulting domain wall characteristics. We have also advanced XRMS methodology by demonstrating how the incorporation of measurements made at incidence angles other than the Bragg angle can be used to resolve a roughness induced ambiguity regarding the effective penetration depth of the X-rays. The analysis confirms the feasibility of engineering a multilayer with either a fixed chiral DW or a hybrid chiral DW by choosing the adequate number of layers, thicknesses and materials. In a HCDW, the part of the DW showing Bloch type magnetization can be finely tuned on purpose by an externally applied in-plane magnetic field, whereas its position within the DW can be tuned by choosing the desired stacking order of the heavy metal, spacer, and magnetic layer set. We have shown that the first order peaks of XRMS measurements (representing the chiral domain structure) are sensitive to inner modifications of the direction of the internal magnetization within the DWs along $\hat{z}$ direction. We also demonstrate how the appearance of a uniformly aligned Bloch-type DW close to the surface is responsible for the “second order peaks” (and their dichroic but symmetric XRMS signal), revealing therefore the hybrid nature of the DWs. This “second order peak” intensity behaves anti-symmetrically with the external field and in a quantitative agreement with the field-dependent behaviour predicted by simulations of XRMS seeded by micromagnetic simulations of the studied samples. By these means we were able to obtain a 3D characterization of the spin textures (HCDW) that are stabilized in these multilayers. It is worth noting that our approach of comparing observed XRMS data with simulations from a proposed 3D magnetic structure (full details in Ref. ^18^) can be applied to any other complex spin textures such as columnar hybrid skyrmions, magnetic bobbers^30^ or magnetic hopfions^31, 32^, both in static and dynamic experiments.

## Methods

### Sample fabrication

All samples are grown on thermally oxidized Si wafers, indicated by ‘$\mid \mid$’ in Table 1. A buffer layer of Ta|Pt or Pt is used to induce a $(1\,1\,1)$ texture, and samples are capped with Pt to avoid oxidation. Details about the growth can be found in Refs. ^18, 24^. The sample S1 is composed of [Pt(1)$\mid$Co(0.8)$\mid$Al$_2$O$_3$(1)]$_{\times 20}$ and its reversed stacking counterpart sample S2 is composed of [Al$_2$O$_3$(1)$\mid$Co(1)$\mid$Pt(1)]$_{\times 20}$. We measure the magnetization at saturation, $M_{\textrm{s}}$, and the effective anisotropy field $\mu _0H_{\textrm{K}} = 2K_{\textrm{eff}}/M_{\textrm{s}}$ using Superconducting Quantum Interference Device (SQUID) and Alternating Gradient Field Magnetometer (AGFM) respectively. Using these values we deduce the effective uniaxial interface anisotropy $K_{\textrm{u}} = K_{\textrm{eff}} + \mu _0 M_{\textrm{s}}^2/2$ (positive $K_{\textrm{eff}}$ means that out-of-plane magnetization is favoured). The exchange stiffness, *A*, is estimated using interpolation of Brillouin light scattering data in a reference multilayer with thicker Co layers (around 1.8 nm) and the effective $A=0$ is found for thickness below $\sim 0.4$ nm^33^. The DMI exchange parameter *D* is deduced from the period of the stripe-domain configuration obtained after in-plane demagnetization^18^. The MFM measurements are performed using a very low moment magnetic tip and a two-pass mode. In the two-pass mode, the first one in alternating contact (often known as “tapping mode”) and the second at some distance from the surface as determined by the first pass.

### XRMS

The level of polarization on both lines are above 99%. An X-rays incidence angle of 15.86± 0.01$^{\circ }$ for sample S1, 15.57 ± 0.01$^{\circ }$ for sample S2, and 16.7 ± 0.01$^{\circ }$ for sample S3 corresponding to the first experimentally determined Bragg peak of the each multilayer stacking. The resonant scattering peaks from the magnetic domain configuration are captured by a CCD detector ($2000 \times 2000$ pixels for ALBA, $2048 \times 2048$ pixels for SOLEIL).

### Simulations

The micromagnetic simulations were performed using MuMax3^34^. For each system S1 and S2, one period of the parallel stripes was modeled, and constituted of two alternated magnetic domains separated by two DWs initialized with intermediate internal angle between Néel and Bloch configurations. For the down branch of the hysteresis, the system is initialized with a saturated in-plane magnetization along $\hat{y}$ direction. Magnetic parameters are indicated in Table 1. The cell size was set at 0.18$\times$1$\times$0.2 nm$^3$ for S1 and for 1$\times$1$\times$1 nm$^3$ for sample S2 along $\hat{x}$, $\hat{y}$ and $\hat{z}$ directions following the definition of Fig. 2a, in order to match the smallest periodicity of the magnetic multilayer within the field range: 131 nm for sample S1 and 100 nm for S2. A field dependent variation of the periodicity experimentally observed was not included in the micromagnetic or XRMS simulations. Periodic boundary conditions were allowed including 32 $\times$ 128 replicas of the system along $\hat{x}$ and $\hat{y}$ directions to model their contributions to the dipolar field distribution^18^. The magnetic field is set in along direction of the stripes ($\hat{y}$ direction), proceeding in steps of 0.01 T from 0.5 to − 0.5 T for sample S1 and 0.3 to − 0.3 T for sample S2, as in the experiments. The magnetization texture was obtained after relaxing the energy [using the MuMax3 function relax()], then running the simulation for a short time (20 ps for S1 and 500ps for S2) at finite temperature (300 K) to overcome remaining energy barriers, and finally minimizing [using the MuMax3 function minimize()] the energy again^35–37^.

## Data Availability

The datasets used and/or analysed during the current study available from the corresponding author on reasonable request.
